# The genome sequence of the Stable Fly, *Stomoxys calcitrans *(Linnaeus, 1758)

**DOI:** 10.12688/wellcomeopenres.23623.1

**Published:** 2025-02-07

**Authors:** Ian Sims, Chris Raper, Olga Sivell, Natural History Museum Genome Acquisition Lab, Darwin Tree of Life Barcoding collective, Wellcome Sanger Institute Tree of Life Management, Samples and Laboratory team, Wellcome Sanger Institute Scientific Operations: Sequencing Operations, Wellcome Sanger Institute Tree of Life Core Informatics team, Tree of Life Core Informatics collective, Darwin Tree of Life Consortium

**Affiliations:** 1 Syngenta International Research Station, Jealott’s Hill, Berkshire, England, UK; 2 Natural History Museum, London, England, UK

**Keywords:** Stomoxys calcitrans, Stable Fly, genome sequence, chromosomal, Diptera

## Abstract

We present a genome assembly from an individual specimen of *Stomoxys calcitrans* (Stable Fly; Arthropoda; Insecta; Diptera; Muscidae). The genome sequence has a total length of 1,070.90 megabases. Most of the assembly (98.96%) is scaffolded into 5 chromosomal pseudomolecules.The mitochondrial genome has also been assembled and is 17.6 kilobases in length. Gene annotation of this assembly on Ensembl identified 15,757 protein-coding genes.

## Species taxonomy

Eukaryota; Opisthokonta; Metazoa; Eumetazoa; Bilateria; Protostomia; Ecdysozoa; Panarthropoda; Arthropoda; Mandibulata; Pancrustacea; Hexapoda; Insecta; Dicondylia; Pterygota; Neoptera; Endopterygota; Diptera; Brachycera; Muscomorpha; Eremoneura; Cyclorrhapha; Schizophora; Calyptratae; Muscoidea; Muscidae; Muscinae; Stomoxyini; *Stomoxys*; *Stomoxys calcitrans* (Linnaeus, 1758) (NCBI:txid35570)

## Background

*Stomoxys calcitrans* (Linnaeus, 1758) is the only species in the genus *Stomoxys* Geoffroy, 1762 occurring in Europe and it is common and widely distributed in Britain ( Chandler, 2024). The species is cosmopolitan ( Duvallet & Hogsette, 2023; Pont, 1973).

It is a medium sized fly, 5.5–7.7mm in length, with greyish body (black base colour covered with dusting). It has four undusted black stripes on the thorax and a pattern of medial triangles on the anterior part of tergites 3, 4 and 5 and two black marks in the posterior part of tergites 3 and 4 one on each side. It can be readily recognised by the long (longer than height of head) and strongly sclerotised proboscis without distinct labella, adapted for blood sucking; palpi short, one third of the length of mentum or approximately the length of the antenna, hypopleuron with hairs on upper part, lower calypter strongly diverging from scutellum ( d’Assis Fonseca, 1968; Taylor *et al.*, 2013).

Adults feed on blood, which is required for reproduction, although they can survive on flower nectar or juices from decaying organic matter ( Hindle, 1914; Jones *et al.*, 1992). They attack horses and cattle, rarely humans, other mammals, birds and reptiles. The long proboscis is equipped with tiny teeth at the end of labium which penetrate the skin by rasping ( Taylor *et al.*, 2013). The bite of *Stomoxys* is painful and causes irritation to cattle and horses. As the common name of this species suggest it is often found in stables and in close proximity to hosts, in the countryside. It is an important pest causing considerable economic loss through reduced weight of livestock and decreased milk production, due to its negative impact on livestock (irritation, blood loss, reduced feeding, diseases) ( Mullens *et al.*, 2006; Taylor *et al.*, 2012). Considerable attention has been given to the control measures of that species ( Foil & Hogsette, 1994; González *et al.*, 2024; Patra *et al.*, 2018; Zumpt, 1973).

The female fly requires blood meals for the ovaries to mature. Once gravid, she lays batches of 25 to 50 eggs in dung or decaying plant material, on which larvae then feed. Pupation occurs in drier substrates. The development of eggs, larvae and pupae depends on temperature (optimal 27 °C) and the complete life cycle takes 12 to 60 days. The adult flies live for about a month ( Taylor *et al.*, 2013). Research on the development of *S. calcitrans* in laboratory conditions has been conducted by Kunz *et al*. (1977), Lysyk (1998) and Gilles *et al*. (2005). Development of the embryo and 1st instar larva has been documented by Ajidagba *et al.* ( 1983, 1985); 1st, 2nd and 3rd instar larvae of *S. calcitrans* were SEM imaged and described by Friesen *et al*. (2015).

*Stomoxys calcitrans* is a mechanical vector of pathogens such as trypanosomes, which cause diseases in horses, cattle, sheep and goats (e.g. surra, mal de caderas, nagana) and spread human African trypanosomiasis. They are also vectors of anthrax, dermatophilosis (also affecting humans) and is an intermediate host of *Habronema* nematodes. All listed diseases can cause a considerable economic loss and some affect humans as well as the livestock ( Baldacchino *et al.*, 2013; Barlaam *et al.*, 2020; Taylor *et al.*, 2013; Zaria, 1993). A summary of pathogens transmitted by *Stomoxys* flies has been provided by Baldacchino *et al*. (2013).

The genome of *Stomoxys calcitrans* was published by Olafson *et al.* (2021) and the phylogeny of the genus *Stomoxys* was studied by Dsouli *et al*. (2011). The high-quality genome of *Stomoxys calcitrans* presented here was sequenced from a single specimen (NHMUK013805957; SAMEA112222201) from Hartslock Nature Reserve, England. The genome was sequenced as part of the Darwin Tree of Life Project, a collaborative effort to sequence all named eukaryotic species in the Atlantic Archipelago of Britain and Ireland. It will aid research on genetics, biology of *S. calcitrans* and phylogeny of *genus* *Stomoxys* and the family Muscidae.

## Genome sequence report

**Figure 1.**
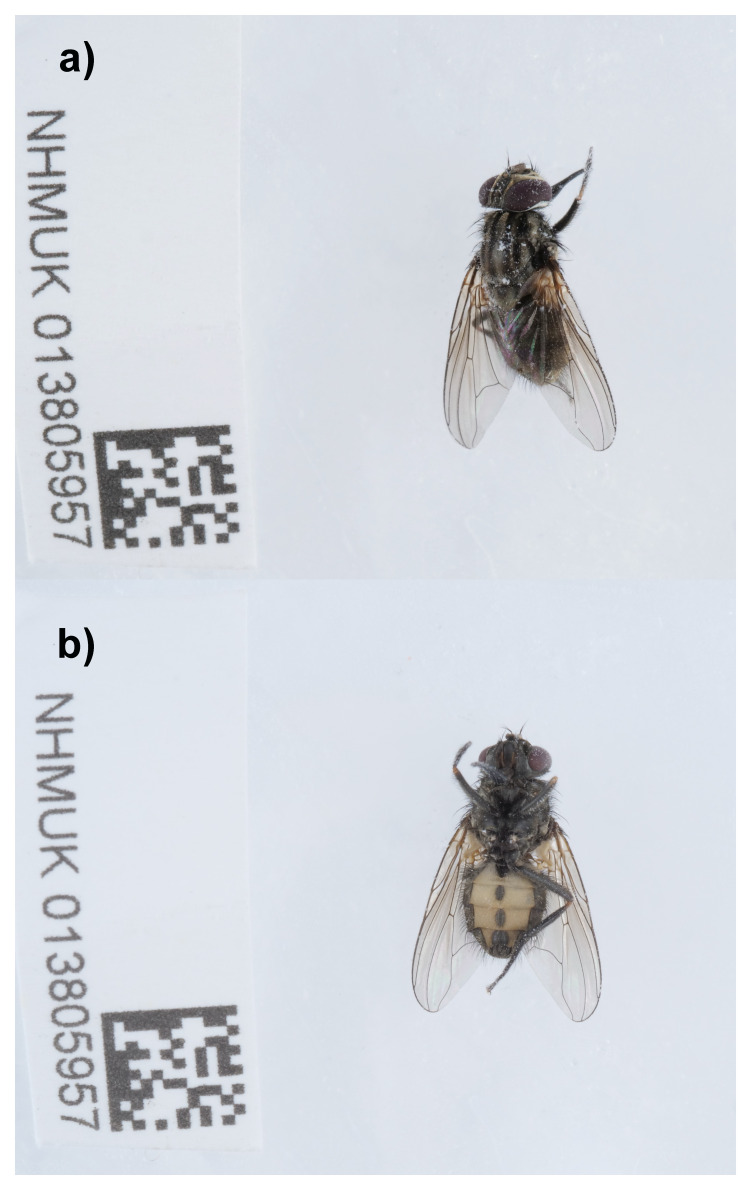
Photographs of the *Stomoxys calcitrans* (idStoCalc2) specimen used for genome sequencing. **a**) Dorsal view and **b**) ventral view.

The genome of *Stomoxys calcitrans* ( Figure 1) was sequenced using Pacific Biosciences single-molecule HiFi long reads, generating a total of 29.82 Gb (gigabases) from 2.59 million reads, providing an estimated 39-fold coverage. Primary assembly contigs were scaffolded with chromosome conformation Hi-C data, which produced 104.65 Gb from 693.04 million reads. Specimen and sequencing details are summarised in Table 1.

**Table 1.** Specimen and sequencing data for *Stomoxys calcitrans*.

Project information			
**Study title**	Stomoxys calcitrans (stable fly)		
**Umbrella BioProject**	PRJEB62559		
**Species**	*Stomoxys calcitrans*		
**BioSample**	SAMEA112222201		
**NCBI taxonomy ID**	35570		
Specimen information			
**Technology**	**ToLID**	**BioSample accession**	**Organism part**
**PacBio long read sequencing**	idStoCalc2	SAMEA112222301	Whole organism
**Hi-C sequencing**	idStoCalc2	SAMEA112222301	Whole organism
Sequencing information			
**Platform**	**Run accession**	**Read count**	**Base count (Gb)**
**Hi-C Illumina NovaSeq 6000**	ERR11496081	6.93e+08	104.65
**PacBio Sequel IIe**	ERR11483512	2.59e+06	29.82

Assembly errors were corrected by manual curation, including 120 missing joins or mis-joins and 15 haplotypic duplications. This reduced the assembly length by 0.6% and the scaffold number by 18.13%. The final assembly has a total length of 1,070.90 Mb in 297 sequence scaffolds, with 1,506 gaps, and a scaffold N50 of 199.7 Mb ( Table 2).

**Table 2.** Genome assembly data for *Stomoxys calcitrans*, idStoCalc2.1.

Genome assembly		
Assembly name	idStoCalc2.1	
Assembly accession	GCA_963082655.1	
*Accession of alternate haplotype*	*GCA_963082745.1*	
Span (Mb)	1,070.90	
Number of contigs	1,804	
Number of scaffolds	297	
Longest scaffold (Mb)	271.96	
Assembly metrics *		*Benchmark*
Contig N50 length (Mb)	1.6	*≥ 1 Mb*
Scaffold N50 length (Mb)	199.7	*= chromosome N50*
Consensus quality (QV)	58.5	*≥ 40*
*k*-mer completeness	Primary: 74.98%; alternate: 72.39%; combined: 98.24%	*≥ 95%*
BUSCO v5.4.3 lineage: diptera_odb10	C:98.7%[S:98.2%,D:0.5%], F:0.3%,M:1.0%,n:3285	*S > 90%*, *D < 5%*
Percentage of assembly mapped to chromosomes	98.96%	*≥ 90%*
Sex chromosomes	Not identified	*localised homologous pairs*
Organelles	Mitochondrial genome: 17.6 kb	*complete single alleles*
Genome annotation of assembly GCA_963082655.1 at Ensembl		
Number of protein-coding genes	15,757	
Number of non-coding genes	6,556	
Number of gene transcripts	33,619	

* Assembly metric benchmarks are adapted from Rhie *et al.* (2021) and the Earth BioGenome Project Report on Assembly Standards September 2024. ** BUSCO scores based on the diptera_odb10 BUSCO set using version 5.4.3. C = complete [S = single copy, D = duplicated], F = fragmented, M = missing, n = number of orthologues in comparison. A full set of BUSCO scores is available at https://blobtoolkit.genomehubs.org/view/Stomoxys_calcitrans/dataset/GCA_963082655.1/busco.

**Figure 2.**
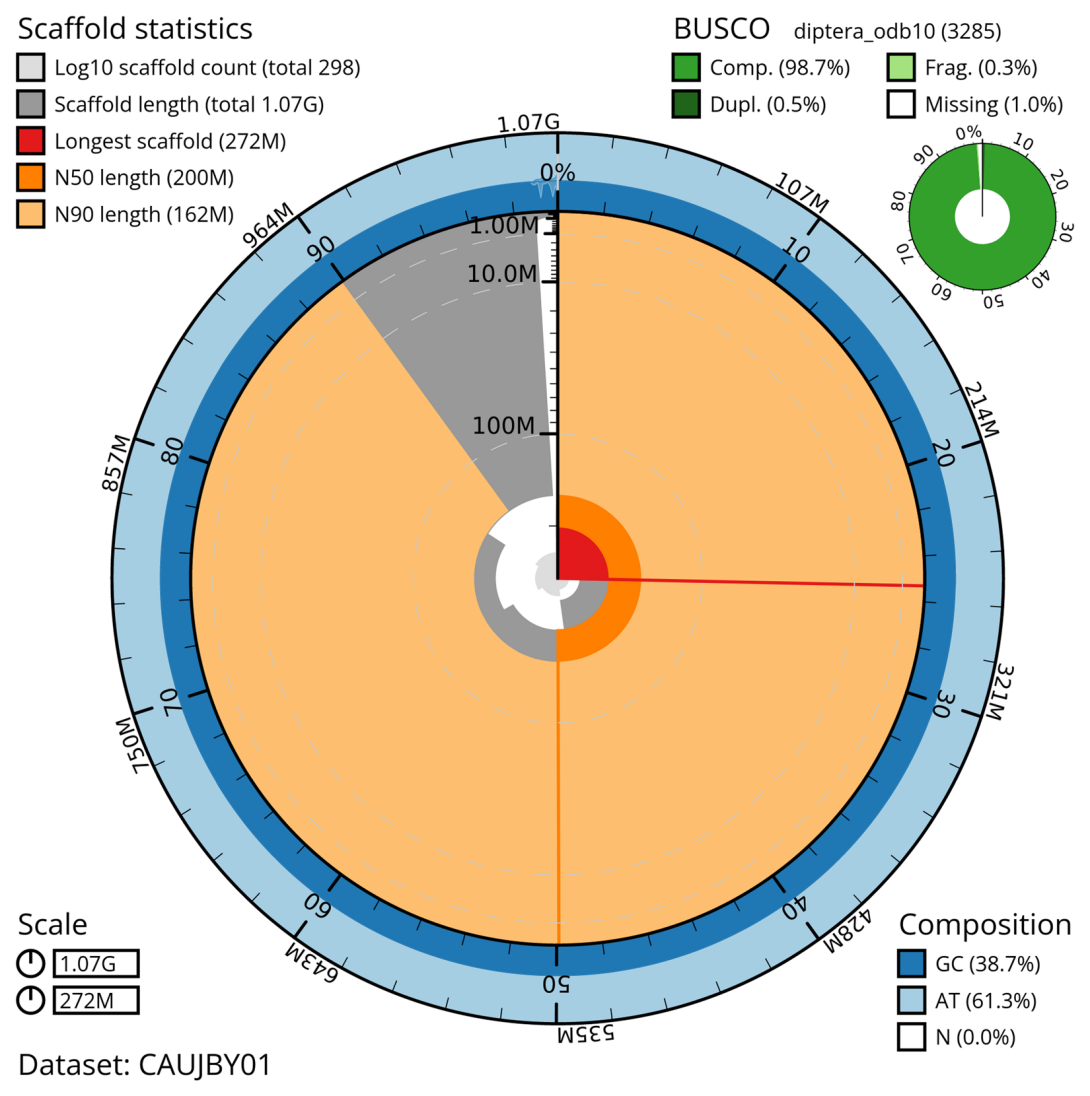
Genome assembly of *Stomoxys calcitrans*, idStoCalc2.1: metrics. The BlobToolKit snail plot provides an overview of assembly metrics and BUSCO gene completeness. The circumference represents the length of the whole genome sequence, and the main plot is divided into 1,000 bins around the circumference. The outermost blue tracks display the distribution of GC, AT, and N percentages across the bins. Scaffolds are arranged clockwise from longest to shortest and are depicted in dark grey. The longest scaffold is indicated by the red arc, and the deeper orange and pale orange arcs represent the N50 and N90 lengths. A light grey spiral at the centre shows the cumulative scaffold count on a logarithmic scale. A summary of complete, fragmented, duplicated, and missing BUSCO genes in the diptera_odb10 set is presented at the top right. An interactive version of this figure is available at https://blobtoolkit.genomehubs.org/view/CAUJBY01/dataset/CAUJBY01/snail.

**Figure 3.**
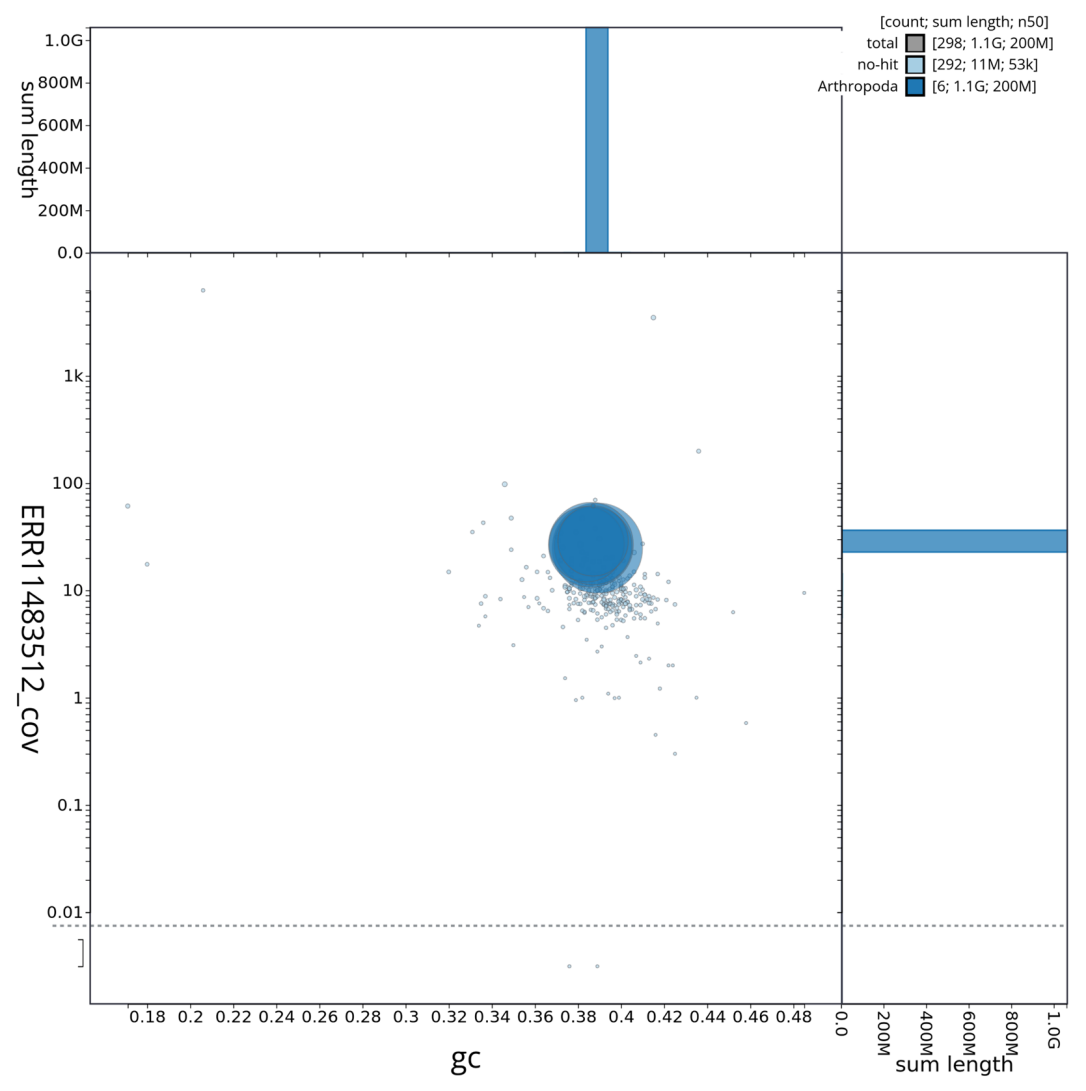
Genome assembly of *Stomoxys calcitrans*, idStoCalc2.1: BlobToolKit GC-coverage plot showing sequence coverage (vertical axis) and GC content (horizontal axis). The circles represent scaffolds, with the size proportional to scaffold length and the colour representing phylum membership. The histograms along the axes display the total length of sequences distributed across different levels of coverage and GC content. An interactive version of this figure is available at https://blobtoolkit.genomehubs.org/view/CAUJBY01/dataset/CAUJBY01/blob.

**Figure 4.**
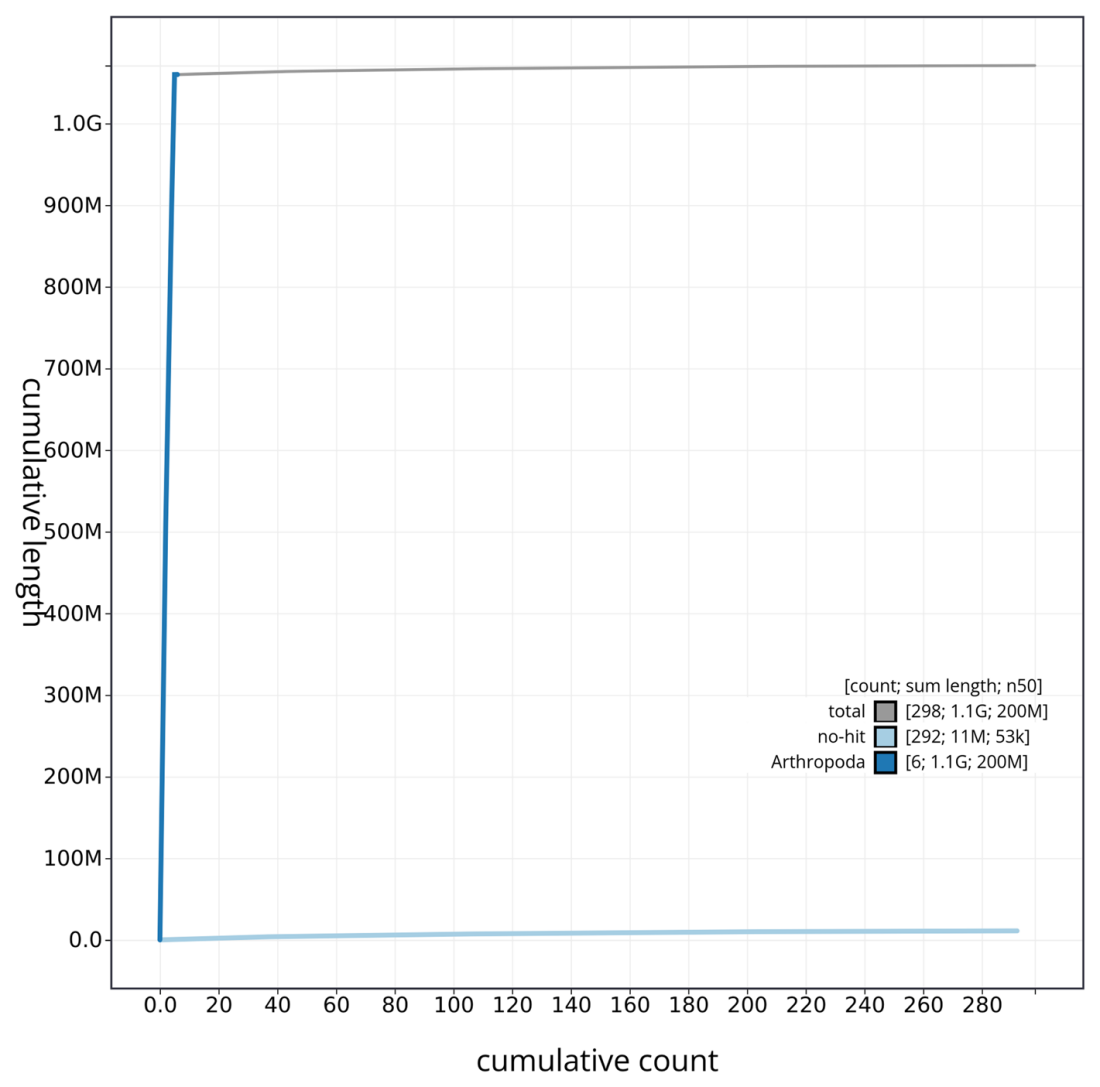
Genome assembly of *Stomoxys calcitrans* idStoCalc2.1: BlobToolKit cumulative sequence plot. The grey line shows cumulative length for all sequences. Coloured lines show cumulative lengths of sequences assigned to each phylum using the buscogenes taxrule. An interactive version of this figure is available at https://blobtoolkit.genomehubs.org/view/CAUJBY01/dataset/CAUJBY01/cumulative.

The snail plot in Figure 2 provides a summary of the assembly statistics, indicating the distribution of scaffold lengths and other assembly metrics. Figure 3 shows the distribution of scaffolds by GC proportion and coverage. Figure 4 presents a cumulative assembly plot, with separate curves representing different scaffold subsets assigned to various phyla, illustrating the completeness of the assembly.

**Figure 5.**
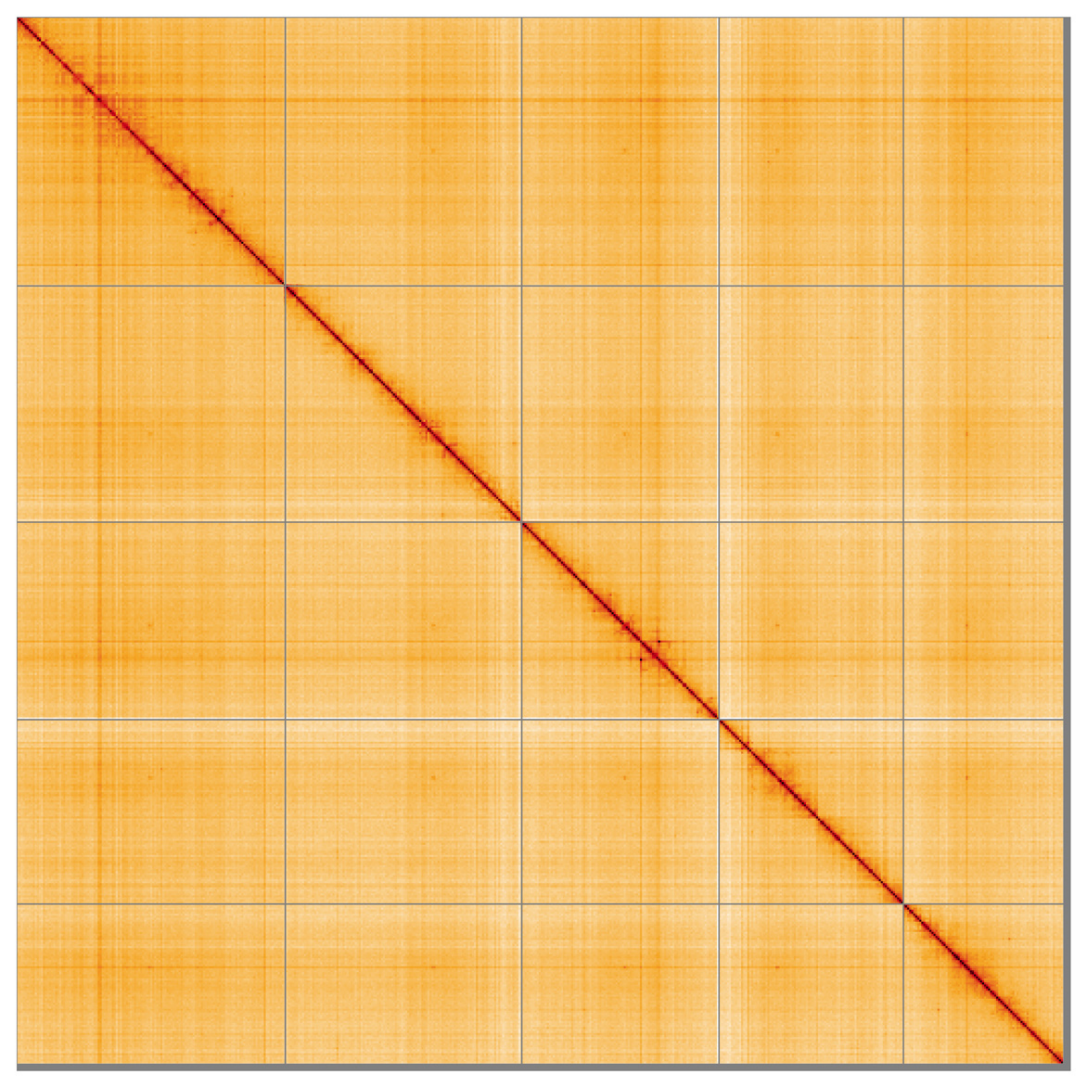
Genome assembly of *Stomoxys calcitrans* idStoCalc2.1: Hi-C contact map of the idStoCalc2.1 assembly, visualised using HiGlass. Chromosomes are shown in order of size from left to right and top to bottom. An interactive version of this figure may be viewed at https://genome-note-higlass.tol.sanger.ac.uk/l/?d=JAPYyKJLTeuZGsgC4yEGzA.

Most of the assembly sequence (98.96%) was assigned to 5 chromosomal-level scaffolds. These chromosome-level scaffolds, confirmed by the Hi-C data, are named in order of size ( Figure 5; Table 3). During manual curation it was noted that the exact order and orientation of contigs on chromosome I (57–67 Mb) is unknown. A sex chromosome could not be identified.

**Table 3.** Chromosomal pseudomolecules in the genome assembly of *Stomoxys calcitrans*, idStoCalc2.

INSDC accession	Name	Length (Mb)	GC%
OY720435.1	1	271.96	39.0
OY720436.1	2	239.16	38.5
OY720437.1	3	199.75	38.5
OY720438.1	4	186.65	38.5
OY720439.1	5	162.29	38.5
OY720440.1	MT	0.02	20.5

While not fully phased, the assembly deposited is of one haplotype. Contigs corresponding to an alternate haplotype have also been deposited. The mitochondrial genome was also assembled and can be found as a contig within the multifasta file of the genome submission, and as a separate fasta file.

The final assembly has a Quality Value (QV) of 58.5 for the combined assemblies. The *k*-mer completeness for the combined assemblies is 98.24% (primary assembly: 74.98%, alternate haplotype: 72.39%). BUSCO (v5.4.3) analysis using the diptera_odb10 reference set ( *n* = 3,285) indicated a completeness score of 98.7% (single = 98.2%, duplicated = 0.5%).

## Genome annotation report

The *Stomoxys calcitrans* genome assembly (GCA_963082655.1) was annotated at the European Bioinformatics Institute (EBI) on Ensembl Rapid Release. The resulting annotation includes 33,619 transcribed mRNAs from 15,757 protein-coding and 6,556 non-coding genes ( Table 2; https://rapid.ensembl.org/Stomoxys_calcitrans_GCA_963082655.1/Info/Index). The average transcript length is 20,557.81. There are 1.51 coding transcripts per gene and 4.51 exons per transcript.

## Methods

### Sample acquisition and DNA barcoding

An adult specimen of *Stomoxys calcitrans* (specimen ID NHMUK013805957, ToLID idStoCalc2) was collected from Hartslock Nature Reserve, England, United Kingdom (latitude 51.51, longitude –1.11) on 2021-07-29, using an aerial net. The specimen was collected by Ian Sims (British Entomological and Natural History Society) and identified by Chris Raper (Natural History Museum) and preserved by dry freezing at –80 °C.

The initial identification was verified by an additional DNA barcoding process according to the framework developed by Twyford *et al*. (2024). A small sample was dissected from the specimens and stored in ethanol, while the remaining parts were shipped on dry ice to the Wellcome Sanger Institute (WSI) ( Pereira *et al.*, 2022). The tissue was lysed, the COI marker region was amplified by PCR, and amplicons were sequenced and compared to the BOLD database, confirming the species identification ( Crowley *et al.*, 2023). Following whole genome sequence generation, the relevant DNA barcode region was also used alongside the initial barcoding data for sample tracking at the WSI ( Twyford *et al.*, 2024). The standard operating procedures for Darwin Tree of Life barcoding have been deposited on protocols.io ( Beasley *et al.*, 2023).

Metadata collection for samples adhered to the Darwin Tree of Life project standards described by Lawniczak *et al*. (2022).

### Nucleic acid extraction

The workflow for high molecular weight (HMW) DNA extraction at the Wellcome Sanger Institute (WSI) Tree of Life Core Laboratory includes a sequence of procedures: sample preparation and homogenisation, DNA extraction, fragmentation and purification. Detailed protocols are available on protocols.io ( Denton *et al.*, 2023b). The idStoCalc2 sample was prepared for DNA extraction by weighing and dissecting it on dry ice ( Jay *et al.*, 2023). Tissue from the whole organism was homogenised using a PowerMasher II tissue disruptor ( Denton *et al.*, 2023a).

HMW DNA was extracted in the WSI Scientific Operations core using the Automated MagAttract v2 protocol ( Oatley *et al.*, 2023). The DNA was sheared into an average fragment size of 12–20 kb in a Megaruptor 3 system ( Bates *et al.*, 2023). Sheared DNA was purified by solid-phase reversible immobilisation, using AMPure PB beads to eliminate shorter fragments and concentrate the DNA ( Strickland *et al.*, 2023). The concentration of the sheared and purified DNA was assessed using a Nanodrop spectrophotometer and Qubit Fluorometer using the Qubit dsDNA High Sensitivity Assay kit. Fragment size distribution was evaluated by running the sample on the FemtoPulse system.

### Hi-C preparation

Tissue from the idStoCalc2 sample was processed at the WSI Scientific Operations core, using the Arima-HiC v2 kit. Tissue (stored at –80 °C) was fixed, and the DNA crosslinked using a TC buffer with 22% formaldehyde. After crosslinking, the tissue was homogenised using the Diagnocine Power Masher-II and BioMasher-II tubes and pestles. Following the kit manufacturer's instructions, crosslinked DNA was digested using a restriction enzyme master mix. The 5’-overhangs were then filled in and labelled with biotinylated nucleotides and proximally ligated. An overnight incubation was carried out for enzymes to digest remaining proteins and for crosslinks to reverse. A clean up was performed with SPRIselect beads prior to library preparation.

### Library preparation and sequencing

Library preparation and sequencing were performed at the WSI Scientific Operations core.

***PacBio HiFi***

At the minimum, samples were required to have an average fragment size exceeding 8 kb and a total mass over 400 ng to proceed to the low input SMRTbell Prep Kit 3.0 protocol (Pacific Biosciences, California, USA), depending on genome size and sequencing depth required. Libraries were prepared using the SMRTbell Prep Kit 3.0 (Pacific Biosciences, California, USA) as per the manufacturer’s instructions. The kit includes the reagents required for end repair/A-tailing, adapter ligation, post-ligation SMRTbell bead cleanup, and nuclease treatment. Following the manufacturer’s instructions, size selection and clean up was carried out using diluted AMPure PB beads (Pacific Biosciences, California, USA). DNA concentration was quantified using the Qubit Fluorometer v4.0 (Thermo Fisher Scientific) with Qubit 1X dsDNA HS assay kit and the final library fragment size analysis was carried out using the Agilent Femto Pulse Automated Pulsed Field CE Instrument (Agilent Technologies) and gDNA 55kb BAC analysis kit.

Samples were sequenced using the Sequel IIe system (Pacific Biosciences, California, USA). The concentration of the library loaded onto the Sequel IIe was between 40 - 135 pM. The SMRT link software, a PacBio web-based end-to-end workflow manager, was used to set-up and monitor the run, as well as perform primary and secondary analysis of the data upon completion.

***Hi-C***

For Hi-C library preparation, DNA was fragmented using the Covaris E220 sonicator (Covaris) and size selected using SPRISelect beads to 400 to 600 bp. The DNA was then enriched using the Arima-HiC v2 kit Enrichment beads. Using the NEBNext Ultra II DNA Library Prep Kit (New England Biolabs) for end repair, a-tailing, and adapter ligation. This uses a custom protocol which resembles the standard NEBNext Ultra II DNA Library Prep protocol but where library preparation occurs while DNA is bound to the Enrichment beads. For library amplification, 10–16 PCR cycles were required, determined by the sample biotinylation percentage. The Hi-C sequencing was performed using paired-end sequencing with a read length of 150 bp on an Illumina NovaSeq 6000.

### Genome assembly, curation and evaluation

***Assembly***

The HiFi reads were first assembled using Hifiasm ( Cheng *et al.*, 2021) with the --primary option. Haplotypic duplications were identified and removed using purge_dups ( Guan *et al.*, 2020). The Hi-C reads were mapped to the primary contigs using bwa-mem2 ( Vasimuddin *et al.*, 2019). The contigs were further scaffolded using the provided Hi-C data ( Rao *et al.*, 2014) in YaHS ( Zhou *et al.*, 2023) using the --break option for handling potential misassemblies. The scaffolded assemblies were evaluated using Gfastats ( Formenti *et al.*, 2022), BUSCO ( Manni *et al.*, 2021) and MERQURY.FK ( Rhie *et al.*, 2020).

The mitochondrial genome was assembled using MitoHiFi ( Uliano-Silva *et al.*, 2023), which runs MitoFinder ( Allio *et al.*, 2020) and uses these annotations to select the final mitochondrial contig and to ensure the general quality of the sequence.

***Assembly curation***

The assembly was decontaminated using the Assembly Screen for Cobionts and Contaminants (ASCC) pipeline (article in preparation). Flat files and maps used in curation were generated in TreeVal ( Pointon *et al.*, 2023). Manual curation was primarily conducted using PretextView ( Harry, 2022), with additional insights provided by JBrowse2 ( Diesh *et al.*, 2023) and HiGlass ( Kerpedjiev *et al.*, 2018). Scaffolds were visually inspected and corrected as described by Howe *et al*. (2021). Any identified contamination, missed joins, and mis-joins were corrected, and duplicate sequences were tagged and removed. The curation process is documented at https://gitlab.com/wtsi-grit/rapid-curation (article in preparation).

***Evaluation of the final assembly***

The Merqury.FK tool ( Rhie *et al.*, 2020) was used to evaluate *k*-mer completeness and assembly quality for the primary and alternate haplotypes using the *k*-mer databases ( *k* = 31) that were pre-computed prior to genome assembly. The analysis outputs included assembly QV scores and completeness statistics.

A Hi-C contact map was produced for the final, public version of the assembly. The Hi-C reads were aligned using bwa-mem2 ( Vasimuddin *et al.*, 2019) and the alignment files were combined using SAMtools ( Danecek *et al.*, 2021). The Hi-C alignments were converted into a contact map using BEDTools ( Quinlan & Hall, 2010) and the Cooler tool suite ( Abdennur & Mirny, 2020). The contact map is visualised in HiGlass ( Kerpedjiev *et al.*, 2018).

The genome was also analysed within the BlobToolKit environment ( Challis *et al.*, 2020) and BUSCO scores ( Manni *et al.*, 2021) were calculated.

Table 4 contains a list of relevant software tool versions and sources.

**Table 4.** Software tools: versions and sources.

Software tool	Version	Source
BEDTools	2.30.0	https://github.com/arq5x/bedtools2
BLAST	2.14.0	http://ftp.ncbi.nlm.nih.gov/blast/executables/blast+/
BlobToolKit	4.3.7	https://github.com/blobtoolkit/blobtoolkit
BUSCO	5.4.3 and 5.5.0	https://gitlab.com/ezlab/busco
bwa-mem2	2.2.1	https://github.com/bwa-mem2/bwa-mem2
Cooler	0.8.11	https://github.com/open2c/cooler
DIAMOND	2.1.8	https://github.com/bbuchfink/diamond
fasta_windows	0.2.4	https://github.com/tolkit/fasta_windows
FastK	427104ea91c78c3b8b8b49f1a7d6bbeaa869ba1c	https://github.com/thegenemyers/FASTK
Gfastats	1.3.6	https://github.com/vgl-hub/gfastats
GoaT CLI	0.2.5	https://github.com/genomehubs/goat-cli
Hifiasm	0.19.8-r587	https://github.com/chhylp123/hifiasm
HiGlass	44086069ee7d4d3f6f3f0012569789ec138f42b84 aa44357826c0b6753eb28de	https://github.com/higlass/higlass
Merqury.FK	d00d98157618f4e8d1a9190026b19b471055b22e	https://github.com/thegenemyers/MERQURY.FK
MitoHiFi	3	https://github.com/marcelauliano/MitoHiFi
MultiQC	1.14, 1.17, and 1.18	https://github.com/MultiQC/MultiQC
NCBI Datasets	15.12.0	https://github.com/ncbi/datasets
Nextflow	23.04.0-5857	https://github.com/nextflow-io/nextflow
PretextView	0.2.5	https://github.com/sanger-tol/PretextView
purge_dups	1.2.5	https://github.com/dfguan/purge_dups
samtools	1.16.1, 1.17, and 1.18	https://github.com/samtools/samtools
sanger-tol/ascc	-	https://github.com/sanger-tol/ascc
Seqtk	1.3	https://github.com/lh3/seqtk
Singularity	3.9.0	https://github.com/sylabs/singularity
YaHS	1.2a.2	https://github.com/c-zhou/yahs

### Genome annotation

The Ensembl Genebuild annotation system ( Aken *et al.*, 2016) was used to generate annotation for the *Stomoxys calcitrans* assembly (GCA_963082655.1) in Ensembl Rapid Release at the EBI. Annotation was created primarily through alignment of transcriptomic data to the genome, with gap filling via protein-to-genome alignments of a select set of proteins from UniProt ( UniProt Consortium, 2019).

### Wellcome Sanger Institute – Legal and Governance

The materials that have contributed to this genome note have been supplied by a Darwin Tree of Life Partner. The submission of materials by a Darwin Tree of Life Partner is subject to the **‘Darwin Tree of Life Project Sampling Code of Practice’**, which can be found in full on the Darwin Tree of Life website here. By agreeing with and signing up to the Sampling Code of Practice, the Darwin Tree of Life Partner agrees they will meet the legal and ethical requirements and standards set out within this document in respect of all samples acquired for, and supplied to, the Darwin Tree of Life Project. 

Further, the Wellcome Sanger Institute employs a process whereby due diligence is carried out proportionate to the nature of the materials themselves, and the circumstances under which they have been/are to be collected and provided for use. The purpose of this is to address and mitigate any potential legal and/or ethical implications of receipt and use of the materials as part of the research project, and to ensure that in doing so we align with best practice wherever possible. The overarching areas of consideration are:

•   Ethical review of provenance and sourcing of the material

•   Legality of collection, transfer and use (national and international) 

Each transfer of samples is further undertaken according to a Research Collaboration Agreement or Material Transfer Agreement entered into by the Darwin Tree of Life Partner, Genome Research Limited (operating as the Wellcome Sanger Institute), and in some circumstances other Darwin Tree of Life collaborators.

## Data Availability

European Nucleotide Archive: Stomoxys calcitrans (stable fly). Accession number PRJEB62559; https://identifiers.org/ena.embl/PRJEB62559. The genome sequence is released openly for reuse. The *Stomoxys calcitrans* genome sequencing initiative is part of the Darwin Tree of Life (DToL) project. All raw sequence data and the assembly have been deposited in INSDC databases. Raw data and assembly accession identifiers are reported in Table 1 and Table 2.

## Author information

Members of the Natural History Museum Genome Acquisition Lab are listed here: https://doi.org/10.5281/zenodo.12159242.

Members of the Darwin Tree of Life Barcoding collective are listed here: https://doi.org/10.5281/zenodo.12158331.

Members of the Wellcome Sanger Institute Tree of Life Management, Samples and Laboratory team are listed here: https://doi.org/10.5281/zenodo.12162482.

Members of Wellcome Sanger Institute Scientific Operations: Sequencing Operations are listed here: https://doi.org/10.5281/zenodo.12165051.

Members of the Wellcome Sanger Institute Tree of Life Core Informatics team are listed here: https://doi.org/10.5281/zenodo.12160324.

Members of the Tree of Life Core Informatics collective are listed here: https://doi.org/10.5281/zenodo.12205391.

Members of the Darwin Tree of Life Consortium are listed here: https://doi.org/10.5281/zenodo.4783558.
