# Supplementary material for: The genome sequence of the Stable Fly, Stomoxys calcitrans (Linnaeus, 1758)
Source: Wellcome Open Res. 2025 Feb 7;10:48. [Version 1] doi: 10.12688/wellcomeopenres.23623.1 (PMC11868752; doi:10.12688/wellcomeopenres.23623.1)
Supplement: Peer review history

# Reviewer report: Reviewer response for version 1

**DOI:** 10.21956/wellcomeopenres.26063.r119590

**Published:** 2025-02-27

**Authors:** Denise Gemmellaro

**Affiliations:** 1 Department of Biological Sciences, Kean University, Union, New Jersey, USA

This article provide an overview of the biology and ethology of *Stomoxys calcitrans *as well as its importance in medico-veterinary entomology. The article then describes the methods and protocols implemented for the sequencing of the genome of this species, citing the sources of the procedures and standards the authors utilized.

Are sufficient details of methods and materials provided to allow replication by others?

Yes

Is the rationale for creating the dataset(s) clearly described?

Yes

Are the datasets clearly presented in a useable and accessible format?

Yes

Are the protocols appropriate and is the work technically sound?

Yes

Reviewer Expertise:

Entomology

I confirm that I have read this submission and believe that I have an appropriate level of expertise to confirm that it is of an acceptable scientific standard.

# Reviewer report: Reviewer response for version 1

**DOI:** 10.21956/wellcomeopenres.26063.r118527

**Published:** 2025-02-26

**Authors:** Savarimuthu Ignacimuthu, Sivasankaran Kuppusamy

**Affiliations:** 1 St Xavier's College, Palayamkottai, Tamil Nadu, India; 2 Entomology Research Institute, Loyola College, Chennai, Tamil Nadu, India

Authors have done the chromosome-level genome sequence of *Stomoxys calcitrans* (Linnaeus, 1758). They assembled a genome and received the comprehensive sequence of 1070.90 Mb in size. They detected 15,757 protein-coding genes and 6,556 non-coding genes and also observed a huge number of 33,619 gene transcripts from the assembled genome.

****

**Minor comments on the manuscripts**

 In the Background of third paragraph, the second sentence can be modified as “Once gravid, the female lays batches”……………

 Fifth paragraph last sentence “phylogeny of *genus”. *The genus should not be in italics.

 The full name of the genus, *Stomoxys calcitrans*, has been used by the authors throughout the text. The full genus name may be used initially, and then it may be abbreviated to *S. calcitrans.*

 Above all, I confirm that the manuscript meets the necessary scientific standard and is suitable for indexing

Are sufficient details of methods and materials provided to allow replication by others?

Yes

Is the rationale for creating the dataset(s) clearly described?

Yes

Are the datasets clearly presented in a useable and accessible format?

Yes

Are the protocols appropriate and is the work technically sound?

Yes

Reviewer Expertise:

Molecular biology

We confirm that we have read this submission and believe that we have an appropriate level of expertise to confirm that it is of an acceptable scientific standard.
